# MicroRNA-125b: association with disease activity and the treatment response of patients with early rheumatoid arthritis

**DOI:** 10.1186/s13075-016-1023-0

**Published:** 2016-06-02

**Authors:** Veronika Hruskova, Romana Jandova, Lucia Vernerova, Herman Mann, Ondrej Pecha, Klara Prajzlerova, Karel Pavelka, Jiri Vencovsky, Maria Filkova, Ladislav Senolt

**Affiliations:** Institute of Rheumatology and Department of Rheumatology, First Faculty of Medicine, Charles University in Prague, Na Slupi 4, 12850 Prague 2, Czech Republic; Faculty of Science Charles University in Prague, Prague, Czech Republic; Technology Centre ASCR, Prague, Czech Republic

**Keywords:** MicroRNA-125b, Early rheumatoid arthritis, Treatment outcome, Disease activity

## Abstract

**Background:**

MicroRNAs (miRNAs) are small RNAs that regulate gene expression by targeting mRNA. It was proved that some miRNAs are significantly deregulated in rheumatoid arthritis (RA). MicroRNA-125b negatively regulates expression of TNF-α, which plays a crucial role in RA pathogenesis. The aim of this study was to determine the treatment outcome of patients with early RA based on the expression of circulating and cellular miR-125b.

**Methods:**

Total RNA was isolated from the plasma and peripheral blood mononuclear cells (PBMCs) of 58 patients with early RA before and three months after treatment initiation and of 54 age- and sex-matched healthy controls (HC). The expression of miR-125b was measured by TaqMan quantitative PCR. The treatment responders were defined as patients achieving remission or low disease activity (28-joint count disease activity score (DAS28) <3.2). Receiver operating characteristic (ROC) curve and stepwise backward multivariable logistic regression analyses of miR-125b expression were used to predict the disease outcome at three and six months after initiation of treatment.

**Results:**

The expression of miR-125b in the PBMCs and plasma of treatment-naïve early RA patients was significantly lower than that of HC and increased significantly after three months of treatment, particularly in responders. However, only the cellular expression of miR-125b was inversely correlated with disease activity. MiR-125b expression in PBMCs was higher in responders than in non-responders after three months (*p* = 0.042). Using ROC analysis, the cellular expression of miR-125b, but not the disease activity at baseline, predicted the treatment response after three months of therapy (area under the curve 0.652 (95 % CI 0.510 to 0.793); *p* = 0.048).

**Conclusion:**

The expression of miR-125b in PBMCs of treatment-naïve patients may present a novel biomarker for monitoring the treatment outcome during the early phase of RA.

## Background

Rheumatoid arthritis (RA), which affects approximately 1 % of the world population, is an autoimmune disease that if not treated effectively is associated with persistent synovitis, leading to severe joint destruction, development of joint deformities, and increased risk of cardiovascular diseases [1]. A targeted therapy approach is an optimal treatment strategy providing the best results for suppressing inflammation, thus avoiding irreversible joint damage and the development of comorbidities in patients with RA [2]. Some patients achieve remission or at least low disease activity shortly after the initiation of treatment with conventional synthetic disease-modifying antirheumatic drugs (cs-DMARDs), whereas others do not respond despite combination therapy or the use of biologic DMARDs. Despite tremendous progress in the treatment of RA, long-term remission in many patients and complete cure remain elusive [3, 4].

One of the factors contributing to a lack of therapeutic response may be the epigenetic regulation of gene expression that seems to escape current targeted therapies [5]. Epigenetic modification is characterized by changes in gene expression without alteration of the nucleotide sequence. This process results from the posttranslational modification of DNA-binding molecules and from the posttranscriptional repression of targeted protein-coding genes [6]. The latter mechanism is mediated by a large number of small non-coding RNAs, including micro-RNA (miRNAs). MicroRNAs can degrade/destabilize targeted mRNA (gene interference) or inhibit protein synthesis and thereby regulate crucial pathways and cellular processes such as cell growth, differentiation, proliferation, and cell death [7, 8]. Deregulation of some miRNAs has been found in many diseases [9, 10], including autoimmune inflammatory disorders such as RA [11–13]. There is evidence that miRNAs can be secreted extracellularly and can be present in plasma or serum in a stable form that is protected from endogenous RNase activity [14]. Furthermore, several circulating miRNAs have already been suggested as potential biomarkers of disease activity in RA [15, 16].

The identification of sensitive biomarkers that predict the response to therapy during the early phases of RA remains challenging. Recently, the circulating miR-125b levels have been associated with chemotherapeutic resistance in breast cancer patients [17], and an elevated serum level of miR-125b has been suggested to be a potential predictive biomarker of the treatment response to rituximab in patients with RA [18]. To date, no study has investigated the expression of miR-125b in patients with treatment-naïve early RA. Therefore, the aim of our study was to determine whether the expression of cellular or circulating miR-125b may predict the outcome of treatment in patients with early RA.

## Methods

### Patients

Fifty-eight patients with early RA who fulfilled the 2010 American College of Rheumatology (ACR)/European League Against Rheumatism (EULAR) classification criteria for RA [19], with a duration of symptoms <6 months were included in this study and were prospectively followed in the Prague Early RA Clinic (PERAC) at the Institute of Rheumatology, Prague, Czech Republic as previously described [20]. The control group consisted of 54 age-matched and sex-matched healthy individuals.

Disease activity was assessed using the 28-joint count disease activity score-erythrocyte sedimentation rate (DAS28-ESR) at baseline and at 3 and 6 months after the initiation of treatment. The patients were categorized into non-responders if they had moderate to high disease activity (DAS28 ≥ 3.2) and into responders if they achieved low disease activity or remission after 3 or 6 months of treatment.

### RNA isolation

Peripheral blood mononuclear cells (PBMCs) were isolated by standard Ficoll (Greiner Bio-one, Leipzig, Germany) density gradient centrifugation, and pellets were snap frozen and stored at –80 °C until analysis. Plasma samples were stored at –20 °C. Total cellular RNA was isolated by miRNeasy Mini Kit (Qiagen, Hilden, Germany) according to protocol. Residual DNA contamination was removed using the RNase-Free DNase Set (Qiagen) as recommended by the manufacturer. Circulating miRNAs were isolated from plasma samples using phenol-chloroform extraction as previously described [16]. Plasma samples were spiked with 25 fmol each of 3 synthetic miRNAs of *Caenorhabditis elegans* origin (cel-miR-39, cel-miR-54, cel-miR-238) (Qiagen) after denaturation with Trizol LS (Thermo Scientific, Waltham, MA, USA) [16]. The concentration of total RNA was measured using NanoDrop 2000 spectrophotometer (Thermo Scientific).

### Reverse transcription and quantification of miRNAs

The expression of miR-125b in PBMCs and plasma samples of all patients with early RA at baseline and at 3 months of therapy was analyzed and compared to the expression in PBMCs and plasma of HC. Overall, 5 ng of total RNA was reverse-transcribed using TaqMan microRNA Assays (Life Technologies) and TaqMan MicroRNA Reverse Transcription Kit (Life Technologies) in a Thermocycler MyCycler (Biorad, Hercules, CA, USA). Next, TaqMan microRNA Assays (Life Technologies) and TaqMan Universal PCR Master Mix, no AmpErase UNG (Life Technologies) were used to quantify miRNA expression by quantitative polymerase chain reaction (qPCR) in a 7900HT Fast Real-Time PCR System (Applied Biosystems, Foster City, CA, USA). Small nucleolar RNA RNU44 for cellular miRNAs or the average of cel-miR-39, cel-miR-54 and cel-miR-238 for circulating miRNAs (Life Technologies) were used to normalize data in PBMCs or plasma samples, respectively. *X*-fold calculation using the delta cycle threshold (dCt) method was used for calculating the relative expression of miR-125b as follows:

$$ {2^{-}}^{\left(CtmiR-125b{\textstyle \hbox{-} }CtRNU44/ average ofcel-miR-39,cel-miR-54, andcel-miR-238\right)} $$

### Statistical analysis

The Mann-Whitney *U* test and Wilcoxon matched-pairs signed rank test were used where appropriate. Spearman’s correlation test and the Fisher transformation were applied for analysis of correlation and partial correlation. Receiver operating characteristic (ROC) curve analysis of miR-125b expression was performed to predict disease outcome, and the area under the curve (AUC) with 95 % confidence interval (CI) was calculated. To confirm the ROC analysis, stepwise backward multivariable logistic regression was performed. Data were presented as the mean (SD) or median (range). *P* values <0.05 were considered statistically significant. GraphPad Prism, version 6 software was used for the statistical analyses.

## Results

### Clinical characteristics

The clinical characteristics of the patients are shown in Table 1. Prior to treatment, 37patients had high disease activity (DAS28 > 5.1), 15 patients had moderate disease activity (DAS28 3.2–5.1), and 6 patients had low disease activity (DAS28 < 3.2). Treatment with cs-DMARDs was initiated in 56 patients at baseline: 49 patients were treated with methotrexate (mean weekly dose of 15 mg at 3 months), 1 patient was treated with leflunomide, 4 patients were treated with sulfasalazine (mean daily dose of 2 g at 3 months), 2 patients were treated with antimalarial drugs (mean hydroxychloroquine dose 200 mg), 1 patient received a combination of methotrexate and sulfasalazine, and 46 patients received glucocorticoids (mean daily dose of 5 mg of prednisone or equivalent at 3 months). Two patients were receiving only glucocorticoids at 3 months either due to liver toxicity (methotrexate was temporarily discontinued) or planned pregnancy (cs-DMARD was not initiated within the 3 months).

**Table 1:** Characteristics of patients with early rheumatoid arthritis (RA) and healthy controls (HC)

	Early RA			HC
Number	58			54
Sex, female/male, *n*	42/16			41/13
Age, years	54.93 ± 16.18			50.87 ± 15.11
Disease duration	<6 months			
Treatment after 3 months				
Glucocorticoids	46			
Methotrexate	49			
Leflunomide	1			
Sulfasalazine	4			
Hydroxychloroquine	2			
Clinical characteristic	Baseline	3 months	6 months	
RF IgM positivity, %	52	NA	NA	NA
Anti-CCP positivity, %	66	NA	NA	NA
DAS28	5.56 ± 1.56	3.05 ± 1.42	2.86 ± 1.25	NA
CRP, mg/l	9.16 (0.30, 152.20)	3.10 (0.21, 23.85)	2.83 (0.24, 44.44)	1.02 (0.22, 4.36)
ESR, mm/h	27.00 (4.00, 107.00)	15.50 (2.00, 54.00)	14.00 (2.00, 74.00)	NA

Data are expressed as the mean ± SD or median (range) according to the distribution. *Anti-CCP* anti-citrullinated antibodies, *CRP* C-reactive protein, *DAS28* disease activity score, *ESR* erythrocyte sedimentation rate, *HC* healthy controls, *NA* not analyzed, *RF* rheumatoid factor

After 3 months of treatment, there was significant reduction in disease activity served (DAS28 decreased from 5.6 ± 1.6 to 3.1 ± 1.4 and C-reactive protein (CRP) decreased from 9.2 (0.3–152.2) to 3.1 (0.2–23.9) mg/l; *p* < 0.001 for all comparisons). After 3 months of treatment, 24 patients were in remission, 12 patients had low disease activity, 18 patients had moderate disease activity, and 4 patients had high disease activity. The improvement continued: after 6 months of treatment, 28 patients were in remission, 15 patients had low disease activity, 12 patients had moderate disease activity, and 3 patients had high disease activity.

### The expression of miR-125b is lower in patients with early RA

**Fig. 1:**
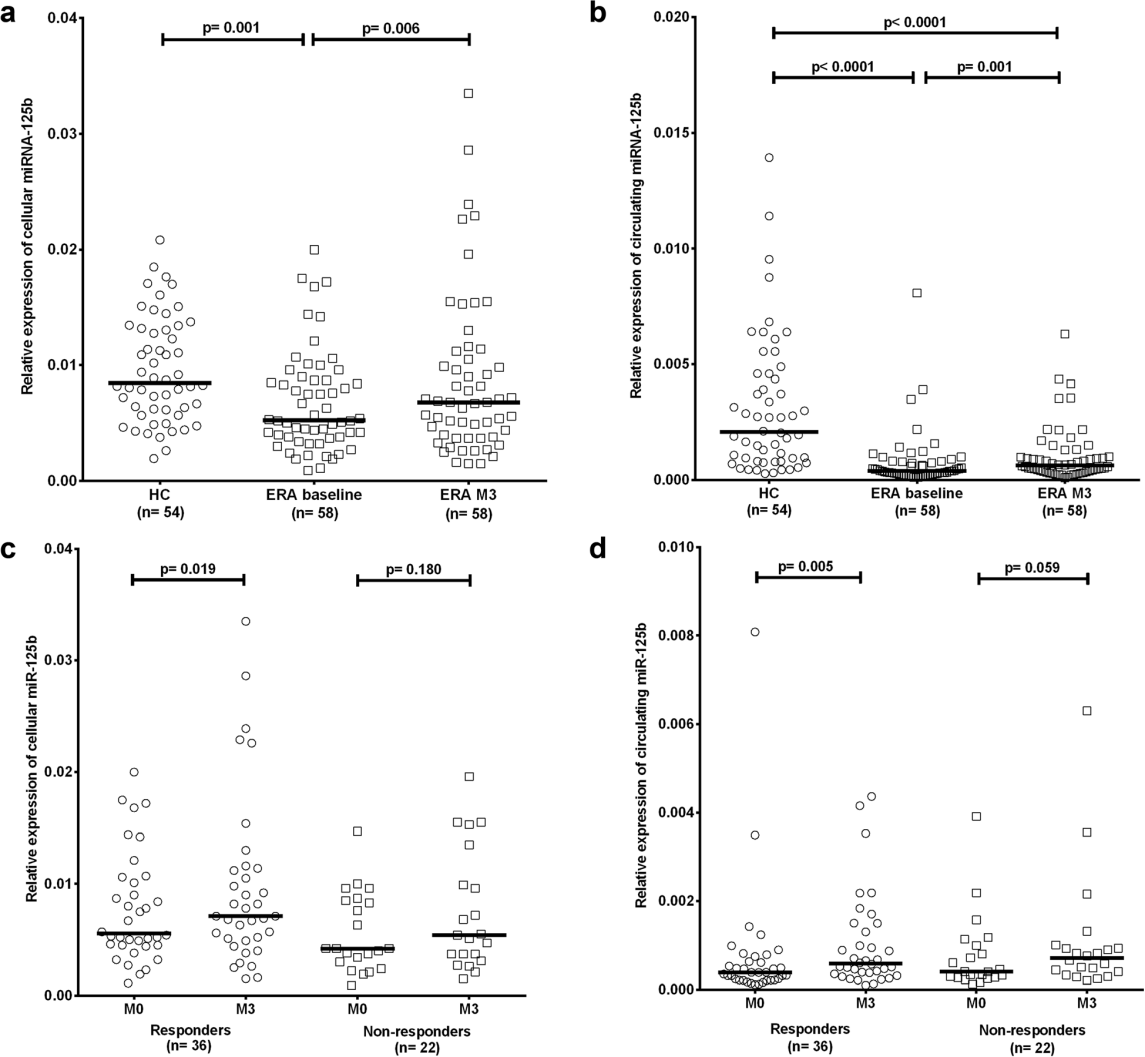
The expression of miR-125b is reduced in peripheral blood mononuclear cells (**a**) and plasma samples (**b**) from patients with early rheumatoid arthritis (*ERA*) compared with healthy controls (*HC*), and significantly increases after 3 months of therapy (*M3*), particularly in patients achieving remission or low disease activity (responders), compared to non-responders (**c**, **d**). *Horizontal line* represents the median. *P* values were estimated using the nonparametric unpaired Mann-Whitney *U* test to calculate differences in the expression of miR-125b between HC and patients with early RA, and the nonparametric paired Wilcoxon test to calculate differences in the expression of miR-125b between patients with early RA before and after therapy

First, we determined miR-125b expression in patients with early RA and in HC and the effect of treatment on the expression of miR-125b. The baseline expression of miR-125b in peripheral blood mononuclear cells (PBMCs) (*p* = 0.001) and in plasma (*p* < 0.0001) was significantly lower in patients with early RA than in HC and increased significantly after 3 months of therapy (*p* = 0.006 and *p* = 0.001, respectively) (Fig. 1a, b). There was a significant increase in miR-125b expression in responders only, compared to patients who did not achieve remission or low disease activity (Fig. 1c, d). Similar changes in the miR-125b expression profile were observed after 3 months in responders and non-responders after 6 months of treatment (*p* = 0.006 and *p* = 0.001, respectively). Increase in miR-125b expression over time was not associated with the dose of glucocorticoids or methotrexate. Furthermore, miR-125b expression in PBMCs also increased in the few responders who were naïve to glucocorticoids (data not shown). However, there was no association between cellular and circulating miR-125b (*r* = –0.222, *p* = 0.094) and no association between the change in cellular or circulating miR-125b and an improvement in disease activity (data not shown).

### Cellular miR-125b is inversely correlated with disease activity

**Fig. 2:**
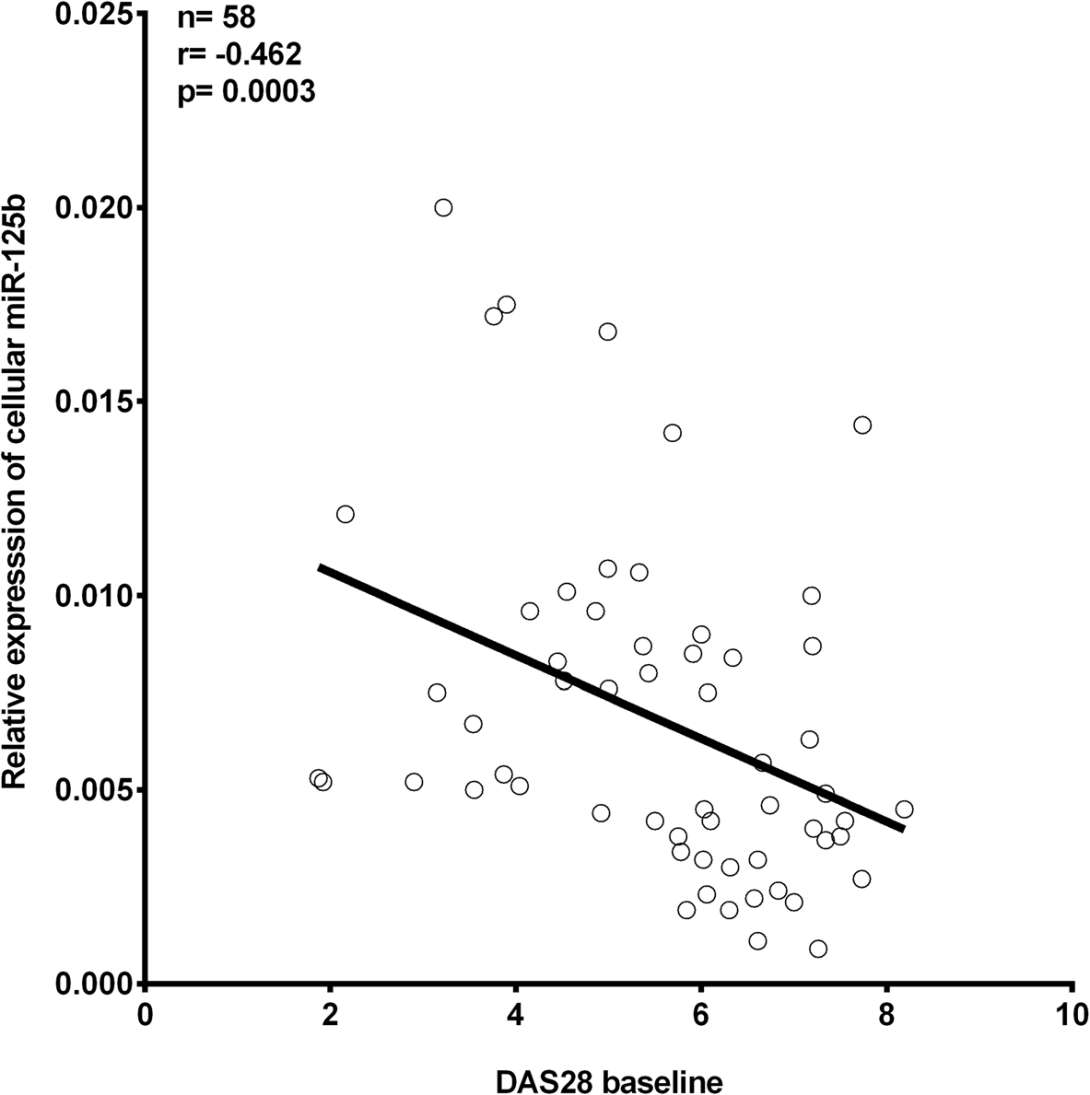
Inverse correlation between the initial expression of miR-125b in peripheral blood mononuclear cells and baseline disease activity assessed by the 28-joint count disease activity score (*DAS28*). Nonparametric Spearman correlation analysis was used to test the association between baseline miR-125b and disease activity

Next, we analyzed whether the expression of miR-125b is associated with disease activity in patients with early RA. We found that the baseline expression of cellular miR-125b was inversely correlated with the DAS28 at baseline (*r* = –0.407; *p* = 0.001) (Fig. 2), with baseline ESR (*r* = –0.375; *p* = 0.003) and with CRP levels (*r* = –0.270; *p* = 0.035). Using age-adjusted analysis, the inverse correlation between miR-125b and disease activity remained significant for DAS28 (*r* = –0.276; *p* = 0.032) and ESR (*r* = –0.268; *p* = 0.042) but not for CRP levels (*r* = –0.194; *p* = 0.137). No such correlations were demonstrated for circulating miR-125b (*r* = 0.117, *p* = 0.384). These data show that lower intracellular expression of miR-125b in PBMCs is present in treatment-naïve patients with early RA, who have higher disease activity.

### Baseline expression of cellular miR-125b predicts treatment response

**Fig. 3:**
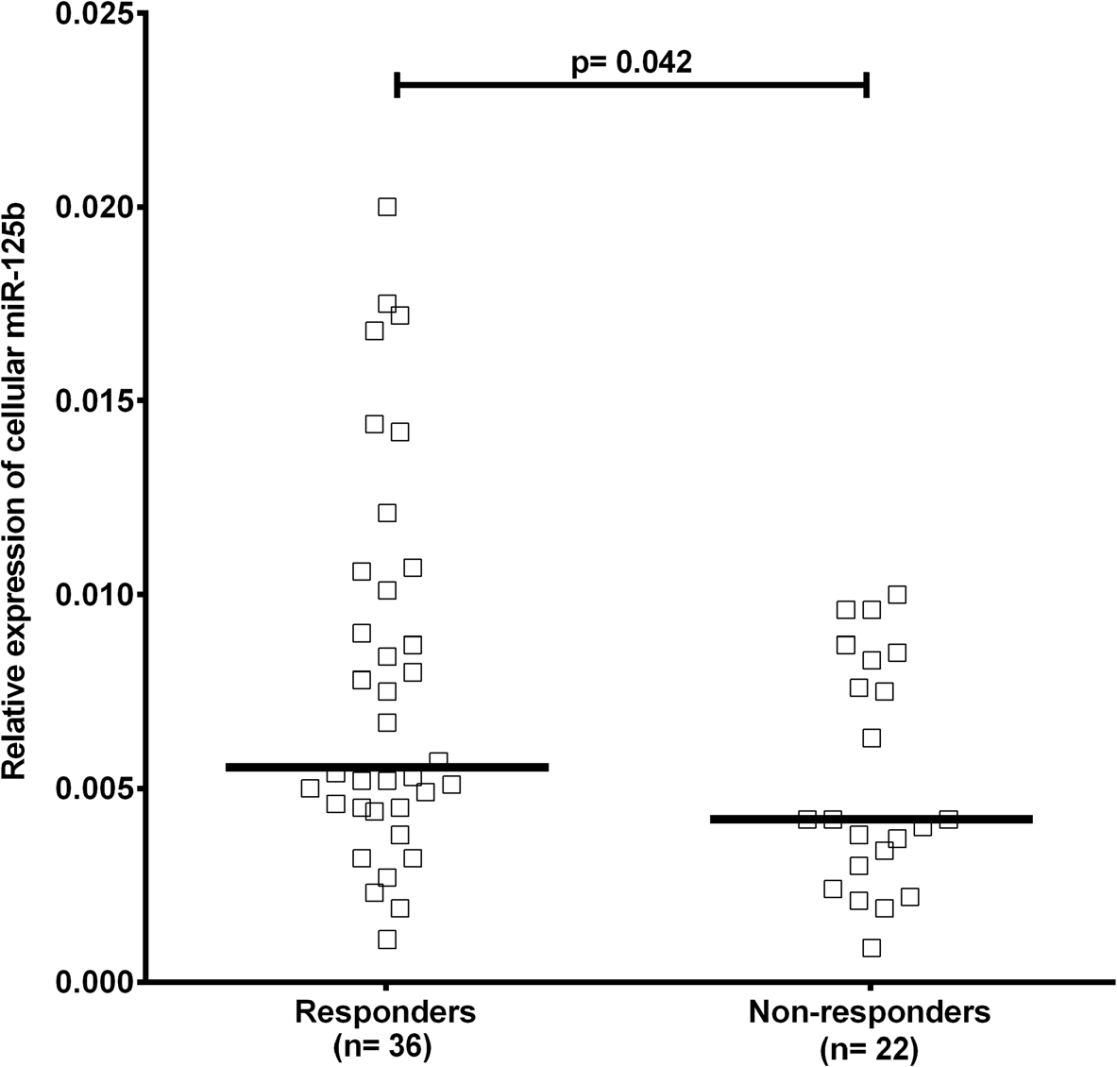
Baseline expression of miR-125b in peripheral blood mononuclear cells is higher in responders than in non-responders. Patients with early rheumatoid arthritis were categorized as non-responders if they had moderate to high disease activity assessed by the 28-joint count disease activity score (DAS28 ≥ 3.2) and as responders if they achieved low disease activity or remission (DAS28 < 3.2) after 3 months of treatment. *Horizontal line* represents the median. *P* values were estimated using the nonparametric unpaired Mann-Whitney *U* test to calculate differences in the expression of miR-125b between responders and non-responders

We also hypothesized that baseline expression of miR-125b may predict treatment response. We demonstrated that baseline cellular (but not circulating) expression of miR-125b was higher in responders to treatment at 3 months than in non-responders (*p* = 0.042) (Fig. 3). However, baseline miR-125b expression did not differ between responders and non-responders to treatment at the 6-month follow up (*p* = 0.321), probably due to the small number of non-responders (n = 15).

**Fig. 4:**
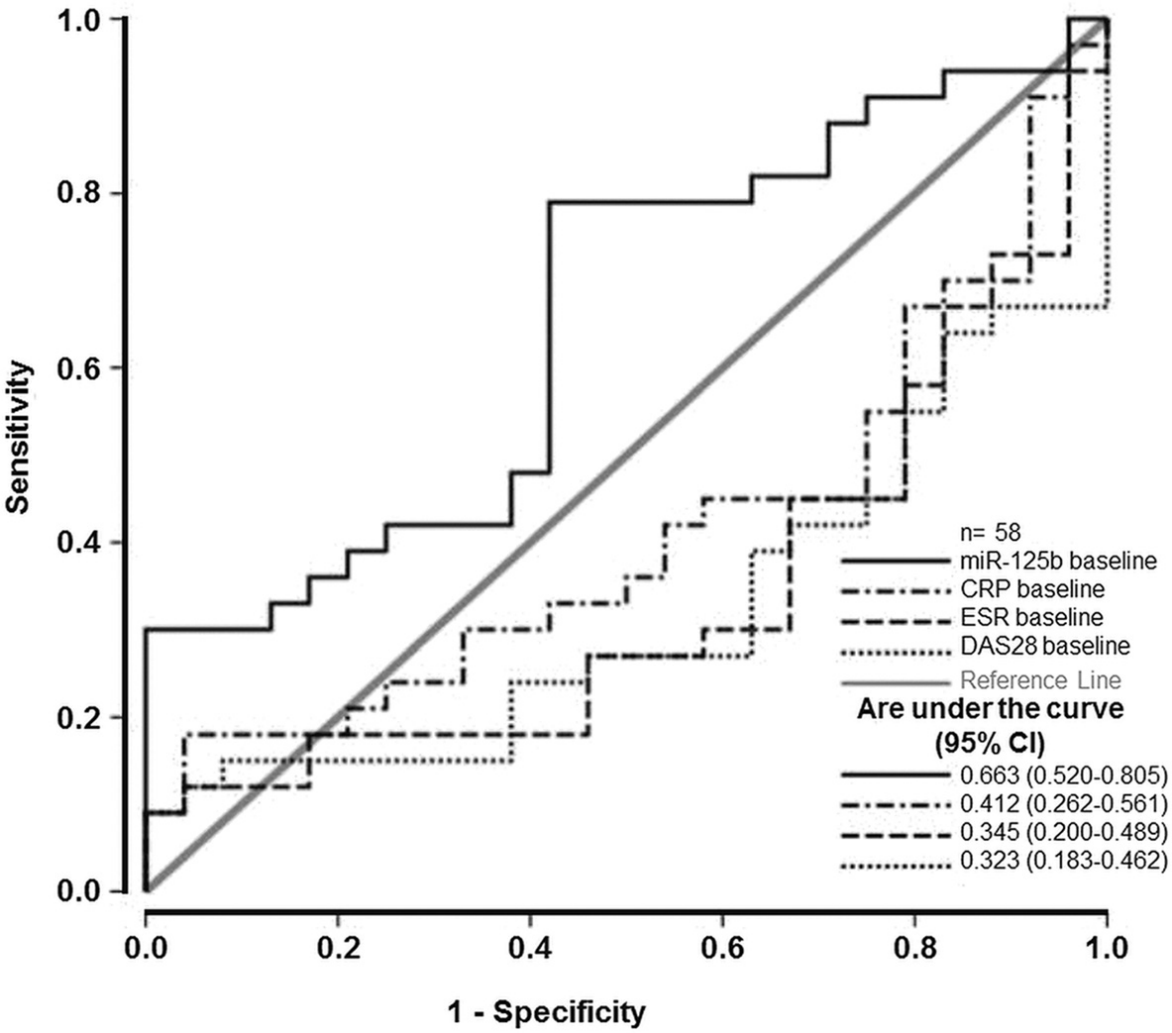
Receiver operating characteristic curve analysis of baseline expression of miR-125b in peripheral blood mononuclear cells as a predictor of achieving optimal treatment outcome (28-joint count disease activity score (*DAS28* < 3.2) after 3 months. Baseline miR-125b expression (*solid black line*) had the highest area under the curve (0.663 (0.520–0.805); *p* = 0.048). C-reactive protein (*CRP*) (*dashed-dotted black line*), erythrocyte sedimentation rate (*ESR*) (*dashed black line*) and DAS28 (*dotted black line*) at baseline are presented

Based on the data showing that baseline miR-125b expression is higher in responders than in non-responders at 3 months, we performed ROC curve analysis to determine the predictive value of baseline miR-125b expression and conventional baseline parameters of disease activity, including DAS28, CRP levels and ESR, in separating patients with early RA who achieve an optimal treatment outcome (remission or low disease activity) from those who do not have such a response. This analysis showed that baseline miR-125b expression in PBMCs was the only predictor of achieving an optimal treatment outcome after 3 months (Fig. 4). The AUC for baseline miR-125b expression was 0.663 (95 % CI 0.520 to 0.805; *p* = 0.048). We then performed stepwise backward multivariate logistic regression analysis of baseline cellular miR-125b expression in combination with DAS28 at 3 months. This analysis confirmed the ROC analysis outcomes and showed that baseline miR-125b expression was a significant and independent predictor of treatment response at 3 months (OR 3.717 95 % CI 1.005 to 13.745; *p* = 0.049).

## Discussion

In the present study, we report that (1) baseline miR-125b expression is lower in patients with early RA than in healthy subjects, (2) cellular miR-125b expression is inversely correlated with RA disease activity, (3) miR-125b expression increased after 3 months of conventional therapy, and (4) higher baseline cellular miR-125b expression predicts the early optimal therapeutic response (DAS28 < 3.2).

The expression of several miRNAs is altered in patients with RA compared to the healthy population [21]. Consistent with previous studies demonstrating reduced expression of miR-125b in some autoimmune diseases, particularly in psoriatic keratinocytes and systemic lupus erythematosus (SLE) CD4+ T lymphocytes [22, 23], we found that the expression of miR-125b in both PBMCs and plasma is lower in treatment-naïve patients with early RA than in HC.

MiR-125b has been described as a negative regulator of TNFα and other pro-inflammatory cytokines such as interferon (IFN)γ, chemokine CCL4 and matrix metalloproteinase (MMP)-13 [24–27]. Thus, we hypothesize that reduced expression of miR-125b could be associated with increased inflammation in RA. However, Duroux-Richard et al. [18] reported increased expression of miR-125b in whole blood and serum from patients with established RA. The discrepancy between this report [18] and our data may be explained by the different material used for miRNA analysis and particularly by the different stages of the disease. It was previously shown that the expression profile of miRNAs is different in whole blood and PBMCs [28]. Moreover, although our study included treatment-naïve patients with RA of short disease duration, Duroux-Richard et al. [18] studied patients with established and long-lasting RA, consistent with our recent study that showed differential expression of circulating miRNAs in early and in established RA [16].

We observed inverse correlation between baseline expression of cellular miR-125b and the parameters of disease activity in patients with early RA. This result is consistent with an inhibitory effect of miR-125b on the expression of pro-inflammatory cytokines, cell proliferation, and apoptosis [24–27, 29]. miR-125b has been inversely associated with age, as its expression is higher in the immune cells of young donors than in older donors [29]. Considering age as a potential confounder, age-adjustment confirmed our data, and the association between baseline miR-125b expression in PBMCs and clinical disease activity remained significant. Furthermore, we demonstrated upregulation of miR-125b in PBMCs from patients with early RA after 3 months of therapy, which was particularly pronounced in responders. This finding may be due to stronger inhibition of pro-inflammatory cytokines, which would lead to a better clinical outcome over time. This hypothesis is supported by a recent observation of increased levels of circulating miR-125b in RA responders to anti-TNFα/DMARD combination therapy [30], though the source of miRNA was not cellular.

Although one study demonstrated upregulation of miR-125b in human B lymphoblast cells after exposure to dexamethasone [31], this upregulation was transient. In our study, we found no association between the dose of glucocorticoids and change in miR-125b expression over time. Furthermore, miR-125b expression in PBMCs also increased in the few responders who were naïve to glucocorticoids. We also did not observe association between the dose of methotrexate and change in miR-125b over time, therefore, it can be speculated that the change in miR-125b expression may not be influenced by the type of therapy, but rather by improvement in disease activity.

To determine the treatment outcome, we performed a predictive analysis by plotting an ROC curve and by logistic regression analysis of the baseline expression of miR-125b and disease activity over time. We demonstrated that higher baseline expression of miR-125b predicts remission or low disease activity after 3 months of therapy. Crucial pro-inflammatory cytokines such as TNFα or IL-6 [24–27, 32] are known downstream targets of miR-125. Therefore, we suggest that the higher cellular miR-125b expression contributes to the achievement of lower inflammatory status and thus, could serve as a biomarker for the early treatment response in patients with early RA. However, no such finding was observed for circulating miR-125b or for miR-125b in PBMCs at the 6-month follow up. We found no correlation between circulating and miR-125b containing PBMCs; therefore, other compartments or mechanisms may influence the levels of circulating miRNAs. Because miR-125b has been demonstrated to be a biomarker of resistance to chemotherapy in pancreatic cancer [32] and circulating miR-125b has been demonstrated to be a predictive biomarker of response to biologic treatment in established RA [18, 30], further studies evaluating the role of miR-125b in RA are needed.

## Conclusion

In conclusion, we have demonstrated that the expression of miR-125b in PBMCs is lower in treatment-naïve patients with early RA and mainly in patients who do not reach the optimal therapeutic outcome at 3-month follow-up. The expression of cellular, but not circulating, miR-125b is inversely associated with RA disease activity and may serve as a potential biomarker of treatment response in early RA.

## Abbreviation

ACR, American College of Rheumatology; Anti-CCP, anti-citrullinated antibodies; AUC, area under the curve; CCL4, C-C motif chemokine ligand 4; CI, confidence interval; CRP, C-reactive protein; cs-DMARDs, conventional synthetic disease-modifying antirheumatic drugs; DAS28, 28-joint count disease activity score; DMARDs, disease modifying antirheumatic drugs; ESR, erythrocyte sedimentation rate; EULAR, European League Against Rheumatism; HC, healthy controls; IFNγ, interferon gamma; IL-6, interleukin 6; miR, miRNA, microRNA; MMP-13, matrix metalloproteinase 13; NA, not analyzed; OR, odds ratio; PBMCs, peripheral blood mononuclear cells; PCR, polymerase chain reaction; PERAC, Prague Early RA Clinic; qPCR, quantitative polymerase chain reaction; RA, rheumatoid arthritis; RF, rheumatoid factor; ROC, receiver operating characteristic curve; SD, standard deviation; SLE, systemic lupus erythematosus; TNFα, tumor necrosis factor alpha.
